# A proposed cure for homosexuality and the circulation of male hormone therapies in Brazil, 1938-1949

**DOI:** 10.1590/S0104-59702025000100023en

**Published:** 2025-05-19

**Authors:** Rodrigo Ramos Lima

**Affiliations:** i Postdoctoral fellow, Department of Preventive Medicine/Faculty of Medicine/Universidade de São Paulo. São Paulo – SP – Brazil contatomagisterio@hotmail.com

**Keywords:** Organotherapy, Homosexuality, Eugenics, Hormones, Endocrinology

## Abstract

This article analyzes *Homossexualismo e endocrinologia* (1938), by the medical examiner Leonídio Ribeiro. The work recommends opotherapy for leading “passive pederasts” towards heterosexuality. Opotherapy, or organotherapy, created in the late 1800s, consisted of using medicines produced from glandular secretions from non-human animals to treat humans with disorders of the endocrine glands. The study examines how the male body was represented and how a culture of compulsory virility was promoted in commercial advertisements for glandular extracts in 1930s and 1940s Brazil. The aim is to contribute to contemporary scholarship on the history of the “gay cure” and its intimate connections with eugenics.

In June 1889, Charles Edouard Brown-Sécquard (1817-1894), a scientist known for his studies in the field of neurophysiology, went to the National Academy of Medicine (Académie Nationale de Médecine) in Paris and announced that he had, for experimental purposes, injected himself with extracts from the testicles of dogs and guinea pigs and felt it had improved his wellbeing and disposition. His research, which aimed to prove it was possible to restore a man’s youthfulness and creative capacity, marked the birth of opotherapy, or organotherapy, which consists of using internal extracts from non-human glands to produce medicines for patients with diseases and disorders of the endocrine glands. The methods employed by Brown-Sécquard were questioned by several physiologists; furthermore, there were doubts as to whether the sex glands were the only ones responsible for the healthy development of human sexuality. Nonetheless, the use of testicular extracts was classified as a therapy that promotes virility and revives male sexuality. For women, chemical preparations from the ovaries of these same animals were indicated for hysteria, uterine problems, and age-related frailty (Borell, 1976).

Rejuvenation became an important chapter in the establishment of endocrinology in Western medicine in the 1920s (Sánchez, 2016; Schlich, 2010). It was at this time that the Austrian scientist Eugen Steinach (1861-1944) developed the notion that men could be rejuvenated by blocking their sperm ducts, since the release of sperm through the vas deferens into the testicle itself would enhance physical capacity and inhibit aging. Vasectomy became synonymous with the absorption of testicular secretions and thus the reinvigorated functioning of the organ. The French surgeon Serge Voronoff (1866-1951) traveled to several countries promoting his monkey testicle grafting technique to treat senility and loss of virility and to restore sexual function. The grafts enabled a surgical form of opotherapy (Sánchez, 2016; Levai, 2016; Cuperschmidt, Campos, 2007). Operating on the sex glands was believed to bring about a revival of sexual and organic vitality (Sengoopta, 2003). However, these testicle transplant procedures differed from contemporary conceptions of organ replacement, insofar as the testicular grafts were used to address not only male sexual issues but also a wide range of diseases (Schlich, 2010).

In recent years, some research has demonstrated the validity of using hormonal technologies and opotherapy to reflect on the history of endocrinology and its contributions to eugenics (Becalossi, 2018, 2020a, 2020b, 2023; Lima, 2021; Logan, 2013; Eraso, 2007; McLaren, 2007). In the Brazilian historiography, organotherapy has emerged in studies related to the development of biotherapeutic products (Lima, 2019; Manzoni, 2013; Benchimol, Teixeira, 1993), gender studies (Rohden, 2009; Lima, 2016), its use in childbirth (Mott, 2002; Nucci, Nakano, Teixeira, 2018), the development of technologies to combat impotence (Levai, 2016; Malcher, 2007), and the trajectory of endocrinology as a specialization (Martins, 2001). According to the anthropologist Fabíola Rohden (2008), in the 1920s the mainstream press carried a range of advertisements for different opotherapeutic products for ovarian function manufactured by Brazilian and international pharmaceutical laboratories. As she explains, the identification of hormones and description of the menstrual cycle in the first decades of the twentieth century put the ovaries center stage in definitions of womanhood: their organic vitality was essential for women to perform their reproductive functions. The entrance of organotherapy into the research agenda involving the female reproductive organs marked a turning point, causing the surgical option of ovariectomy to be shunned in favor of hormone therapies (Rohden, 2008). However, following the assertions made by Nelly Oudshoorn (1995) in her acclaimed study of the history of hormones in Europe, Rohden (2008, p.147) supported, when it came to male hormone therapies, that their sale and “the attempt to create a clinical entity similar to the menopause” both failed.

This article adds a new dimension to this interpretation. As Benninghaus (2012) shows, feminist gender studies in the 1980s, taking a social constructivist approach, contributed to a dearth of research on the history of male sexual health. Chandak Sengoopta (2001) points out that although human testicles were not the object of compulsory medical control in the same way that human ovaries were, research on male gonads must be considered in its own clinical dimension.

This study examines how the therapeutic indications for the treatment of homosexuals proposed in the 1938 tome *Homossexualismo e endocrinologia* (Homosexualism and Endocrinology) can help us shine a light on opotherapeutic practices in Brazil. Although the development of procedures to create purified synthetic hormones in the 1930s meant many organotherapeutic products stopped being used in clinical practice in other countries, the Brazilian case demonstrates that the circumstances in this country favored the continued sale of organotherapeutics alongside the incipient industrial production of synthetic hormones. We argue that the publication of advertisements for the main opotherapeutic products that claimed to restore virility and male sexual health would indicate that there was no such decline in sales of glandular extracts given the prolific and diversified sale and clinical use of testicular extracts designed to address male sexual issues during the 1930s and 1940s.

## A plan to cure homosexualities

The medical examiner Leonídio Ribeiro (1893-1976) became well known in medical circles in Brazil as the first physician to run the Civil Police Identification Institute of the Federal District, in 1931, creating a criminal anthropology laboratory as an annex. The journal *Arquivos de medicina legal e identificação*, which he published and edited between 1931 and 1940, became the main platform for disseminating the criminological work he and his colleagues performed, which included studies on the relationship between black people and crime, the blood purity of the Guarani indigenous group, and deformities of the fingers and toes caused by leprosy (Dias, 2015; Cunha, 2002).

In 1938, Ribeiro published a collection of his texts from earlier in the decade under the title *Homossexualismo e endocrinologia*. The book contained photographs of people detained for homosexuality and new chapters in which he called for the pathologization of homosexuality and, therefore, its decriminalization.^1^ Previous to this, Ribeiro had divulged the conclusions of his biotypology studies with pederasts^2^ in legal and medical journals and at meetings of medical societies. This work had earned him and his team of researchers the Lombroso Prize^3^ from the Royal Academy of Italy, establishing his name, according to Arthur Ramos (cited in Dias, 2015), as the young multiplier of forensic medicine in Brazil.

According to the positivist conception of criminology propounded by the Italian physician Cesare Lombroso (1835-1909), crime was the outcome of the degenerate and atavistic constitution of delinquents. Lombroso encouraged the investigation of hereditary data and morphological features that might indicate any signs of organic imbalance or pathology capable of motivating “abnormal” acts (Dias, 2015). Positivist criminology was multidisciplinary, and endocrinology became the theoretical component that formed the basis of its arguments, providing grounds for the formulation of such concepts as “criminal endocrinopath” (Oliveira Jr., 2012). Ribeiro’s belief that endocrine-related traits catalyzed the formation of criminal constitutions made him one of the few scientists who still defended Lombroso’s ideas at a time when biodeterminist theses were beginning to decline. Even while accepting psychoanalytic explanations, Ribeiro used a range of scientific experiments to argue that the functional imbalance of the internal secretion glands was a causative element of “sexual inversion.” Opotherapy was needed if homosexuality was to be cured.

The preface to *Homossexualismo e endocrinologia* (1938) was written by the Spanish endocrinologist Gregório Marañón (1887-1960), who was chiefly responsible for providing the theoretical and etiological basis of the term “homosexualism” (Dias, 2015). For Marañón, the “*varón-tipo*” and the “*hembra-tipo*” – heterosexual men and women – displayed the characteristics of intersexuality and a range of sexual manifestations that defied medicine. He argued that sexual pathology was indicated by an absence of well-defined sexual traits and a predominance of heterosexual elements. In this sense, sex and sexuality were synonymous. His criteria for diagnosing sexual normality depended on the observation of primary anatomical signs (related to the genitals) and secondary anatomical signs, which were linked to bodily characteristics unrelated to reproduction, such as the distribution of hair, fat, and hip measurements. Functional sexual characteristics were synonymous with the social interpretation of sex, resulting in a biologization of traditional social gender roles. Women’s biological determinants included their maternal instinct, their innate sensitivity to emotional stimuli, and an ineptitude for creative and abstract work, in addition to passive resistance and a high-pitched voice. Men’s most notable features were their instinct for social action, their defense of rationality, and their inclination to work outside the domestic environment (Ferla, 2004).

For Marañón, any “perversion of instinct” was not the direct or only outcome of intersexuality, but a predisposition, insofar as environmental factors ultimately molded an individual’s sexuality. This predisposition was, he argued, awakened from its constitutional slumber when the individual had their first sexual impressions in childhood and during the crisis of puberty, which was when intersexual disturbances came to light and the choice for future sexual normality or abnormality occurred (Ribeiro, 1938). This conceptual maneuver was enabled by the intersections between endocrinological knowledge and neo-Lamarckism, in which the influences of the environment on the ability to alter the constitution of the being, whether through hereditary order or through the influence of hormones on behaviors, were evoked by Eugen Steinach and his followers (Logan, 2013).

Another key reference in *Homossexualismo e endocrinologia* was the Italian endocrinologist Nicola Pende (1880-1970), considered the main founder of biotypology – the science responsible for creating categories of human biotypes (Becalossi, 2020b; MacMillan, 2013; Gomes, 2012). Closely linked to the corporatism of Italian fascism, biotypology provided a theoretical and biological grounding for Benito Mussolini’s government in its desire to produce a “new man” (Dias, 2015, p.114).

In his 1923 work *Le applicazioni dell’endocrinologia allo studio dei criminali: la scuola positi*, Pende had related endocrinology with criminology. By bringing up this reference, Ribeiro was pointing out that Pende conceived of homosexuality as a psychic and somatic syndrome that resulted from a malfunction of the endocrine glands, which differed from the prevailing perception that it was the result only of a malformation of the sexual glands. Accordingly, Ribeiro proposed that homosexuality was caused by the pathological hyperfunction of the thymus after puberty, especially when combined with constitutional hyperthyroidism. He also argued that “disorders of a sexual nature” stemmed from a functional imbalance of the adrenals, which he understood as producing two substances, one medullary and the other cortical, the former acting on the chromaffin system through adrenaline and the latter acting directly on the formation of the genital organs (Ribeiro, 1938).

Chiara Becalossi (2018) shows that the activities of the Institute of Biotypology in Genoa and the main texts published by Nicola Pende in the 1920s meant that opotherapy was used to correct functional imbalances in the glands, which were understood to be hereditary. These putative qualities of opotherapy made it a fitting tool for eugenics. Following Lorenzo Benadusi’s discussion, Becalossi understands that biotypology had at least two eugenic purposes: to promote fertility and to improve Italy’s population stock by stimulating virility and repressing homosexual behavior.

Repressing homosexual behavior was one of the goals of Mussolini’s fascist State, for which purpose it submitted homosexual bodies to opotherapy, phototherapy, controlled diets, and psychotherapy. In his medical treatises, Pende described several cases of pituitary, thyroid, and adrenal gland transplant surgery in patients with sexual impotence and sexual neurasthenia.

For his part, Leonídio Ribeiro resumed the tradition of experimenting with testicular implants on accident victims and men returning from military service with the aim of demonstrating that the patients regained their normal sexual capacity after opotherapy involving testicular grafts:

There is a recent observation by Dartigues, from Paris, of a 33-year-old neuropath, whose old homosexual tendencies were soon improved, with sexual desire and the desire to marry even appearing two months after the transplant operation. Indeed, today ovarian grafts are very common in surgical practice, with excellent results. There are other published observations in which the same success has been achieved. ‘Marañón mentions three cases in which he recommended a graft, according to Voronoff’s theory,’ and in two of them the improvements were quite significant (Ribeiro, 1938, p.171; emphasis in the original).

Ribeiro commented that some sectors of the scientific community harbored reservations regarding the prophylactic use of grafts, explaining that “opotherapy is not, however, a definitively resolved issue, with some authors still doubting its potential” (Ribeiro, 1938, p.171). The creation of standardized tests to identify the efficacy, safety, and quality of glandular extracts was eclipsed as industry gained interest in the synthetic hormones created over the course of the following decade. In 1923, Edgar Allen and Edward Doisy had announced the location, extraction, and partial purification of ovarian hormones, called estrogens (Sterling, 2006). In 1928, the German scientists Selmar Aschheim (1878-1965) and Bernhard Zondek (1891-1966) had published *Immature mouse genital system changes when exposed to pregnant human urine*, in which they explained the physiological roles of folliculin and hormones released by the anterior lobe of the pituitary gland in the development of the sexual gonads. These scientists identified that eggs fertilized by sperm released a hormone called human chorionic gonadotropin (hCG), which releases the signal for the uterus to interrupt the menstrual cycle and prepare the uterine region for pregnancy. This resulted in the creation of the first pregnancy tests. Then, ovarian glandular extracts began to be tested on animals. Amid interest in the commercial potential of these drugs, synthetic female hormones, obtained by extracting estrogen from the urine of pregnant mares and women, were first purified in 1929.

In 1931, the German biochemist Adolf Butenandt (1903-1995) obtained fifty milligrams of male hormone from 25,000 liters of human urine from police barracks. Four years later, Ernest Laquer, working at the European manufacturer Organon, isolated a hormonal product from bull testicles, which he named testosterone. In view of the difficulty of obtaining large enough quantities of these inputs, the solution was to produce new hormonal compounds from organic elements with a similar chemical structure. In 1935, Butenandt, in collaboration with Schering-Kahlbaum, and Leopold Ruzicka, in partnership with Ciba Corporation, simultaneously reported methodologies for transforming cholesterol into synthetic testosterone. These scientists shared the Nobel Prize in Chemistry in 1939 (McLaren, 2012; Gaudillière, 2005). The standard method adopted to measure the effectiveness and quality of the new synthetic male hormones was the monitoring of comb growth in castrated roosters after injection with testosterone (Sterling, 2006). In Brazil, Schering S.A., Ciba Ltda., and Laboratórios Raul Leite were the only three companies that marketed synthetic male hormones in the 1930s.

Curiously, *Homossexualismo e endocrinologia* contains no indication for the use of synthetic male hormones. While the Spanish endocrinologist Gregório Marañón, cited in the work, favored a two-pronged therapeutic approach, associating grafts with opotherapy, Ribeiro (1938, p.174) recommended testicle grafts in association with the ingestion of testicular fluids, preferably fresh, to revitalize sexual function. In addition, he recommended “injections of blood serum, extracted from young animals of the same species and, therefore, rich in testicular hormones.” The objective of early treatment was to diagnose hypogenitalism (i.e., the underdevelopment of the genitals) as proof that homosexuality was linked to the full development of the gonads. In a clear demonstration of appreciation for Serge Voronoff’s techniques, Ribeiro (1938, p.174) reported the following:

Grafts performed on humans using the Voronoff technique, from glands extracted from superior monkeys, although not yet completely efficient in all cases, already demonstrate the possibility of favorable results in certain individuals, especially when performed early. Bauer himself, who highlighted the failures of several authors, in this sense, recognizes their indisputable effectiveness, when conducted before puberty.

Ribeiro turned to his professional mentor, the medical examiner Afrânio Peixoto (1876-1947), author of *Los missexuales* (1931), to justify his planned cure for homosexuals. For Peixoto, homosexuals suffered from a double tuberculosis of the tissues, which had to be treated. With the graft of “one of a young man’s ectopic testicles, he loses his inverted gestures and is wedded” (Ribeiro, 1938, p.93). Another striking reference in *Homossexualismo e endocrinologia* is the argument by the Spanish criminologist Jimenez de Ásua (1889-1970) that the punishment meted out in cases of “deviant” sexuality was “absurd” because the transplantation of genital glands served as a “well-directed opotherapeutic medical treatment” (Ribeiro, 1938, p.55). During the First World War, the Austrians Eugen Steinach and Robert Lichtenstern (1874-1952), the latter a urologist, transplanted the testicle of a heterosexual man into a thirty-one-year-old homosexual soldier suffering from tuberculosis. Twelve days after surgery, the patient reported heterosexual inclinations, six weeks later he experienced his first sexual activity, and soon afterwards he got married. The scientists reported, however, that the patient continued to display behaviors considered effeminate (Schlich, 2010).

When Ribeiro (1936) gave a lecture to the Brazilian Society of Criminology, he was asked by a member of the audience, made up of physicians and legal professionals, whether arresting homosexuals was not an act of violence. He replied that what distinguished his research from that of others was that he made photographic records and quantitative biotypological analyses of passive pederasts, as his therapy only targeted these bodies. On the pathologization of homosexuality, James Green (2003, p.209) explains that those who were effeminate (“faggots”) were always assumed to take the role of a “passive” partner (the one who was penetrated) and that this sexual “passivity” was stigmatized and associated with the “inferior social position of women.” Meanwhile, the “active pederast,” whose public and, presumably, private role was that of the man responsible for penetration, escaped these labels, as a “real” man was granted the possibility of having sexual relations with other men without this harming his social status as a man. After all, treating “active pederasts” and offering treatment for them could open the door to acknowledging an association between “active” male homosexuality and heterosexuality. In other words, the “abnormality” of these individuals should not resemble the desired normality, so as not to reveal any similarity between the penetrative sex of “active pederasts” and the behavior of heterosexual men (Lima, 2016).

The hermeneutics of *Homossexualismo e endocrinologia* is expanded when we compare the broad history of medical and pharmaceutical interests with the rise of endocrinological knowledge and therapies in Brazil between the late nineteenth century and the early decades of the twentieth century. Unlike other experts, Ribeiro did not mention any testicular opotherapeutic products of his preference. Rather, his work reflected a considerable influence of the 1920s medical literature, especially Voronoff’s teachings, expounded to the Brazilian medical profession at the medical conferences of 1928 (Levai, 2016; Cuperschmidt, Campos, 2007).

It is now important to retrace the use of opotherapeutic products in medical clinical practice and in the successive attempts to find a “gay cure.” The following outlines the main products available on the Brazilian pharmaceutical market in the 1930s and 1940s, observing how the social roles expected of men were represented in a wealth of detail in advertisements for the products indicated for the treatment of impotence, neurasthenia, and lack of virility.

## Glandular extracts on shelves, in handbooks, and in physicians’ offices

The population decline caused by the First World War (1914-1918) and Spanish flu (1918) meant any proposals designed to reverse this trend received considerable support. In this context, sexual impotence came to be considered a clinical condition that had social consequences. Furthermore, men were also under pressure to overcome the competition of women and young people on the job market. Sex hormones were held to stimulate physical, emotional, and sexual vigor, reaching an industrial scale. Eugenics, endocrinology, and all treatments aimed at rejuvenating the human species embodied the overriding desire of the twentieth century to overcome the biological limitations expressed in the notions of aging, death, and impotence (McLaren, 2007).

Public policies aimed at improving the hygiene of pregnant women in Brazil were interpreted as a symbol of progress and, above all, a precondition for the success of positive eugenics, which consisted of promoting the biological reproduction of the species and a strengthening of scientific motherhood (Stepan, 2005; Freire, 2008; Becalossi, 2023). In its discourse, endocrinology framed pregnancy as the main cycle responsible for the creation of what Renato Kehl (30 ago. 1930) called “glandular heritage,” namely, the core of hereditary hormonal influences that defined aspects of the life and personality of unborn children. Endocrinology also gained credibility from the success it achieved in treating cases of severe cretinism in children.^4^

In Brazil, an upsurge in the manufacture, by public and private entities, of glandular extracts for the treatment of sexual disorders of the male and female body prompted a growing need to ensure that the sexual excesses associated with the Brazilian constitution were replaced by expressions of sexuality based strictly on the biological function of sex; i.e., the reproduction of the species (Oliveira, 2012). Concerns about male “homosexuality” therefore had strong eugenic overtones because the goal was to promote virility in men so they could marry women and contribute to the biological reproduction of the species (Lima, 2016).

The number of Brazilian publications devoted to discussing the minutiae of sexual behavior from the perspective of normality versus pathology grew in the interwar period. Carrara and Russo (2002) highlight the work of two physicians who described themselves as sexologists and garnered considerable popularity in the country’s medical and scientific community: Hernani de Irajá (1894-1969), from southern Brazil, and José de Albuquerque (1904-1984), specialized in andrology. Both had experience of opotherapeutic products in their clinics, but took quite different positions on them.

José de Albuquerque became known for his advertisements in Rio newspapers of special treatments for male sexual issues, including a cure for “impotence in young men.” However, he was against making extensive use of opotherapeutic products (Albuquerque, 12 mar. 1936). According to Malcher (2007), he collected statistics on the use of testicular opotherapy for “male sexopathies” reported by his patients. Between April 1933 and February 1935, 90% of the men who came to him with complaints of a sexual nature had been instructed to consume testicular and prostate medicines. Of the 2,634 patients, 1,861 had previously consulted other professionals and 1,676 had a prescription for opotherapy. In other words, only 15% of the patients were suitable for “glandular extract therapy,” 75% had no need for such products, and 10% had not been prescribed opotherapeutic products. Albuquerque actively opposed the testicular products and advocated for the founding of andrology in Brazil. However, his activism was not enough to curb the interests of the manufacturers of these hormonal extracts or to prevent the population from accessing them.

In contrast, Hernani de Irajá (1933), in his *Tratamento dos males sexuais* (Treatment of sexual maladies), reported an extensive therapeutic arsenal for the treatment of various sex-related conditions in men and women, followed by a few experiments. For Irajá testicular and prostate hormones were responsible for virility. In his treatment of one homosexual over a four-month period, he had used electrotherapy and testicular, supra, and perirenal extracts, summing 120 injections of interstitial hormone, plus doses of prostate extracts, breathing exercises, vibrating massages in the thyroid area, treatment of constipation, psychoanalysis, and education of the patient’s “will, a change of environment, [and] reading of novels of heterosexual passions” (Irajá, 1933, p.212). Despite the failure of his efforts – only twelve of the ninety-three cases were, he claimed, a success – Irajá did not lose hope of curing homosexuals, reporting that a physician he trusted had cured a lesbian with thyroid extracts (p.255).

Alongside hormonal approaches, Irajá (1933, p.29) reinforced the role of food in the treatment of impotence and emphasized that homosexuals rarely wanted to be cured. Those “tempted by passive sodomy” or “compensation pederasty,” such as sailors, soldiers, and prison inmates, should seek “heterosexual re-education” and endeavor to develop an aesthetic appreciation of the opposite sex through painting and sculpture. They should also move away “from the centers where the inclination has been initiated and developed” and undergo “moral and civic education, psychoanalysis, electrotherapy, and surgery.” He supported the use of electrotherapy, as “big sessions in a long series of positive, high-frequency galvanization muted the anorectal hypersensitivity of a sixteen-year-old boy who engaged in passive pederasty due to the high erogeneity of the region, with damage to the erectogenic zones common in his sex. There were no intestinal parasites” (Irajá, 1933, p.147). According to Irajá, this patient had gone to the clinic spontaneously and was “being healed completely.”

Irajá also recommended the careful observation of children’s bodies, suggesting that in cases of “constitutional inversions without transformation of primary or secondary sexual characteristics, it is a good idea to observe children from the age of three or four.” Parents should also prevent boys and girls from coming into contact with toys from the “opposite sex” (Irajá, 1933, p.148). In the second edition of *Tratamento dos males sexuais*, Irajá (1937) described the clinical symptoms of manifest testicular dystrophy, characterized by “softening of the testicles, softening of the scrotum, lack of erection or incomplete erection with large veins in the penis,” which he treated with testicular juices, penile and testicular diathermy with special electrodes, infrared and ultraviolet rays, heat therapy, and sexual cupping. In addition, he highlighted the importance of supplementing the treatment with injections of “Pansexol (M),” produced by Professor Austregésilo, as well as Anterophysin and cortical, available from “Hormônios Sexuais – masculino S.A.,” Testogan, and others (Irajá, 1937, p.173).

The second edition reported on five case studies of patients with “sexual inversion:” three men and two women. A.F., aged twenty-one, had gynecomastia and “sexual inversion.” His treatment consisted of galvanic electrical stimulation, inserting the negative pole into the penis and the positive pole into the anus, as well as 120 injections of testicular and prostate extracts, physical exercise, massages in the thyroid region, and psychoanalysis for four months. Irajá admitted that his attempt to cure A.F. failed (Becalossi, 2020b, p.19).

The second case of male sexual inversion was a twenty-nine-year-old with a “normotype athletic” build, body hair, masculine voice, and normal sexual glands, who had since childhood worn his sisters’ clothes. In three months of uninterrupted treatment, the young man received 51 hormonal treatments, combined with psychoanalysis and hypnosis. For Irajá, the results were encouraging because although the man still felt desire for other men, he began to have sexual relations with women. The third case of “male sexual inversion” was that of a sixty-eight-year-old man with an “almost normal” constitution, who, despite suffering from hemorrhoids, was unable to avoid having sex with men. His treatment consisted of twice-daily immersion baths of the genitals in water at 45 degrees, in addition to exposing the erogenous zones to ultraviolet light and galvanic electrical stimulation. He was also given testicular, prostate, and renal cortex extracts for three months. Again, the treatment regimen failed (Irajá, 1937).

Another patient was L.S. aged 25, who had female “sexual inversion” and presented typically male behaviors, such as an inclination towards business and mathematics. She had not developed secondary female sexual characteristics. Since the age of ten she had been sexually attracted to women, and she had begun active and passive sexual practices with women at the age of seventeen. L.S. was given forty injections of “gynhormon 50 u.r.” and twenty of pituitary gland extracts. She gained weight with this treatment, but her homosexual inclinations remained unchanged. Subsequently, Irajá added daily sessions of diathermy to the hips to stimulate the ovaries.

Another patient was a forty-year-old woman who had started her sexual activity at age eighteen and had since had sexual relations with several women. The first phase of treatment consisted of fifteen injections of thyroid extracts. Then, thirty more injections of this hormone were given along with a strict rest regimen. As the patient’s physical condition improved, Irajá introduced testicular extracts to the treatment. However, the patient’s homosexual inclinations remained unaltered. According to Becalossi (2020b, p.19), Irajá was skeptical about hormonal therapy because although it rendered improvements to the patients’ bodies, this was not accompanied by a disappearance of homosexual urges.

Sexual neurasthenia was also used to frame sexual behavior deemed deviant. Conceived by the New York neurologist Georges Miller Beard (1838-1883), in 1869, this disease was held to consist of a range of symptoms, such as general fatigue, spinal sensitivity, generalized hyperesthesia, localized and peripheral numbness, dull and sharp (nerve) pain, temporary paralysis, gastric disorders, headaches, sleep problems, pressure in the head, poor mental control, concentration problems, irritation, phobias, sexual disorders such as involuntary ejaculation and partial or complete impotence, irritation of the urethra in men, and displacement of organs, inflammation, and irritation of the uterus and ovaries in women (Zorzanelli, 2009).

**Figure 1:**
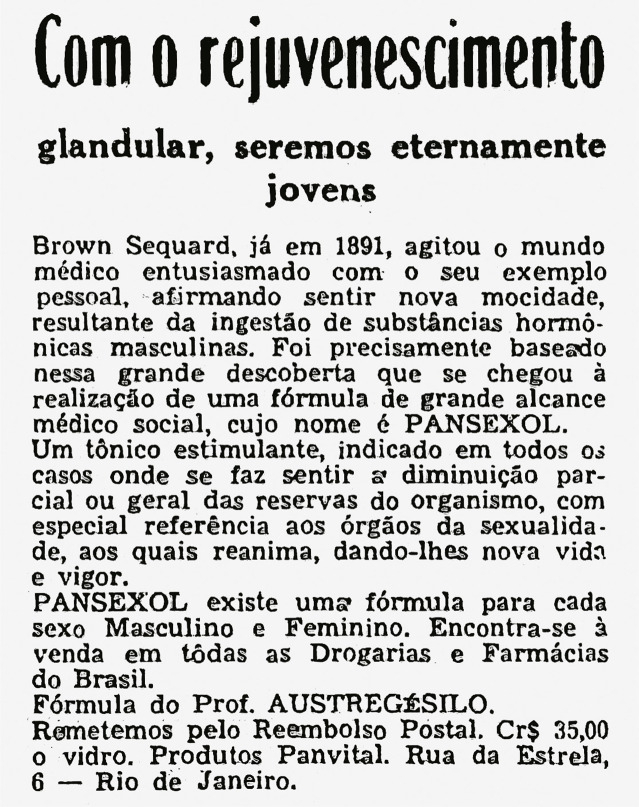
: Advertisement for Pansexol (Com o rejuvenescimento…, 10 abr. 1947)

A good example of the use of neurasthenia as a diagnostic category can be found in *Psiconeuroses e sexualidade: a neurastenia sexual e seu tratamento* (Psychoneuroses and sexuality: sexual neurasthenia and its treatment), published in 1919 by the prestigious psychiatrist Antônio Austregésilo (1876-1960), to whom Irajá dedicated his work. In his work, Austregésilo (1919) recommended the African plant yohimbine in association with strychnine, phosphoric acid, and testicular extracts, known on the market as “sequardine, Poehl’s spermine, Orchidan,” for the treatment of sexual issues in men. With this prescriptive guide, Austregésilo created the formula for Pansexol, a new product made from bull testicle extracts marketed under the intriguing slogan “with glandular rejuvenation, we will be forever young.” The presence of Pansexol on the pages of newspapers (Figure 1) even after the rise of synthetic hormones allows us to infer that the emergence of these new hormones did not bring about an abrupt end to the use of opotherapeutic products. Pansexol was the only glandular extract composed of animal organs advertised in the Rio de Janeiro press until 1962.

Another noteworthy product was a glandular extract prepared by the German sexologist Magnus Hirschfeld (1868-1935) and the andrologist Bernhard Schapiro (1888-1966). Initially sold as Testifortan, this extract was responsible for the majority of the revenues of the Institute for Sexual Science, in Germany. From 1929, it was marketed as Titus Pearls (Pérola Titus) (McLaren, 2007). It was popular in Brazil in the 1930s, appearing in different newspapers that circulated in the capital city (Rio de Janeiro) and several states across the country. In addition to referencing the “Famous Berlin Institute,” its advertisements featured a range of bold designs. The press releases about the product expounded in persuasive terms on male “sexual insufficiency or disorder.” Made from testicular extracts associated with pituitary and adrenal gland extracts, Pérola Titus, it was claimed, would restore male glandular functions and reinstate men’s “normal possibilities of perpetuating the species” (Para os grandes…, 28 maio 1933).

The advertisements for Pérola Titus focused on the values of heroism, strength, and virility, claiming that “all men should be selfless, courageous, and heroic.” To attain such heights, men must have “a perfect endocrine glandular system” (Heróis, set. 1935, p.3). A young man with endocrine dysfunction could be considered “an old man, to all intents and purposes,” while an old man with “active and balanced glands” could be considered “virtually young.” The focus of the advertisements was to warn of the signs of neurasthenia, such as an “erratic, volatile and tempestuous nature,” oscillating with traits of meekness, inertia, fatigue, sexual asthenia, and calmness (O mal…, set. 1941).

**Figure 2:**
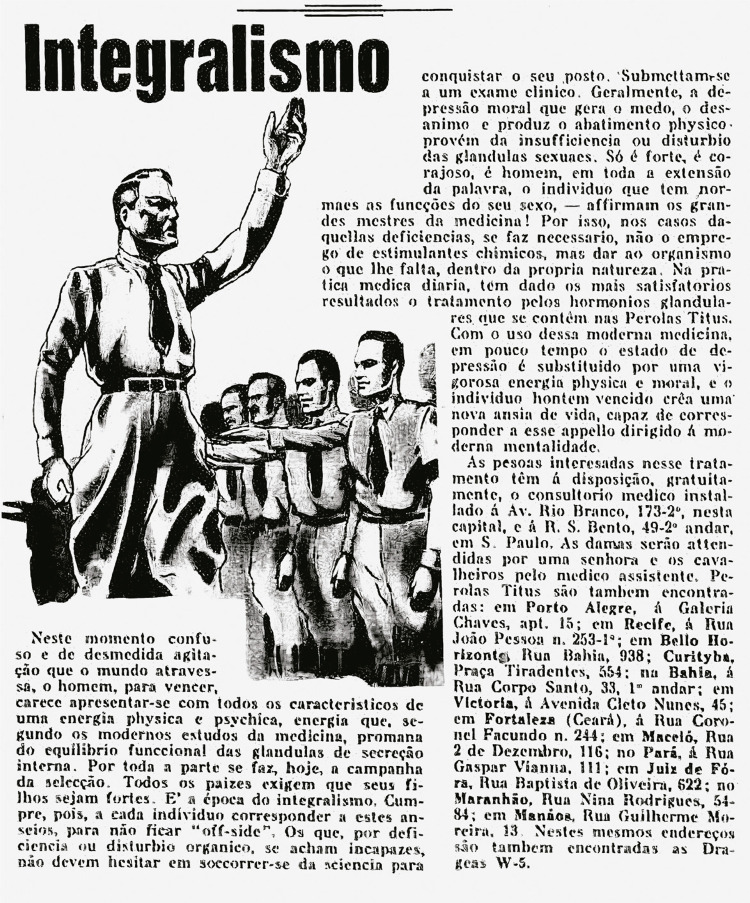
: Advertisement for Pérolas Titus (Integralismo, 19 set. 1933)

The advertisements also invoked the political sentiment that was prevailing at the time both domestically and abroad, expressed in the growth of integralist movements and far-right political parties and an intense appreciation for all things military. The “Integralism and Sexual Dictatorship” advertising campaign (Figure 2) claimed that to “beat” this sense of “excessive agitation,” men should ensure their internal secretion glands were in harmony. Given that there were widespread “selection campaigns,” where different countries “requir[ed] their children to be strong,” the advertisement served as a kind of call for hormonal enlistment.

Also sold in Rio de Janeiro’s pharmacies were “Drágeas Hormônicas” (hormone pills), which were advertised as “a blow to neurasthenia.” These pills, produced by Professor Francisco Figari, from the Royal University of Genoa, were composed of organic phosphorus and hormones from “certain internal secretion glands,” and were claimed to have the capacity to repair damage caused by sexual neurasthenia due to their “more efficient, perhaps more stable” restorative powers “than the famous graft recommended by Voronoff” (Drágeas…, 6 jan. 1937). Consumers keen to find out more about these hormone tablets could obtain a monograph on them, which was distributed free of charge at the “Department of Publications of Scientific Neotherapy, on Rua do Ouvidor.”

Also intended for treating glandular problems were Gotas Mendelianas (“Mendelian Drops”), which were sold in pharmacies and advertised in the Rio de Janeiro press. These drops would, it was claimed, activate the “germinating [glands] in men” and the glands “in women’s ovaries” and would stimulate the nervous system. According to the advertisements, these drops did not affect the heart and were “valuable” and “irreplaceable” for people who needed “a rapid, invigorating nerve tonic.” They would dispel organic malaises as if by “magic” and would give “new life and courage to old neurasthenics, making them strong and potent” (Gotas…, 29 nov. 1938).

**Figure 3:**
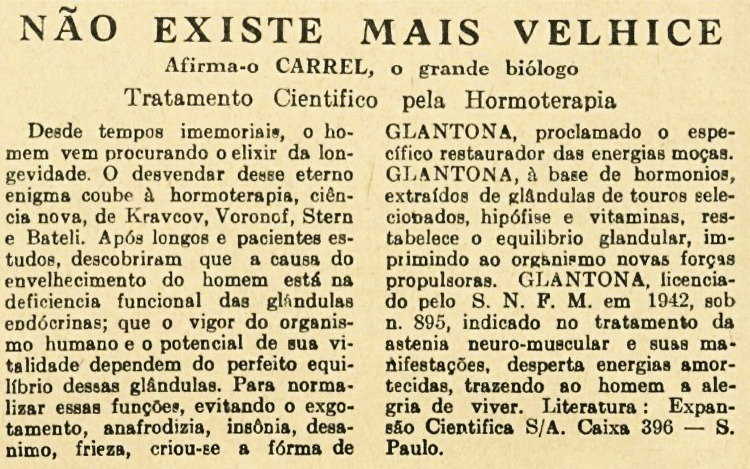
: Advertisement for Glantona (Não existe…, 5 mar. 1949)

Another product sold in pharmacies was Glantona (Figure 3), a glandular extract made from bull’s testicles held to be able to cure sexual disorders such as impotence, sexual neurasthenia, premature senility, and physical and mental frailty, in addition to combating fatigue and premature aging. It was claimed to preserve and replenish the vigor of youth by nourishing certain glands associated with the reproductive sphere (Fraqueza…, 14 jun. 1937).

In the early 1930s, purified synthetic hormones began to be sold in Brazil by the multinationals Schering and Ciba Ltda. and by the Brazilian company Laboratórios Raul Leite. New synthetic drugs started to appear in specialized medical journals and mainstream newspapers, and were produced on a growing scale by the Brazilian pharmaceutical industry between the 1940s and 1960s. The drivers of this growth were mostly prescriptions for the treatment of reproductive sterility in men and women and for female contraceptives (Lima, 2021; Bonan, Teixeira, Nakano, 2017).

Despite José de Albuquerque’s concerted militancy against opotherapeutic practices and the emerging discourse among endocrinologists and physiologists in favor of synthetic hormones, testicular opotherapeutic remedies continued to appear in newspapers and medical manuals addressing male sexual issues throughout the 1930s and 1940s. These advertisements did more than just reveal the pharmaceutical industry’s expectations *vis-à-vis* men’s social, political, and biological behavior; they expressed the idea that glandular dysfunctions should not stop men from engaging in sexual activity, as it was a man’s responsibility to engage in such activities and take measures to boost their health. The eugenic overtones in the calls for the reproduction of the species and the contributions of endocrinology to the care of male and female sexual health stimulated a diverse supply of glandular extracts in different Brazilian states.

## Final considerations

The dynamics of the prevention, control, and repression of deviant male sexuality penetrated Brazil’s public health policies at the turn of the twentieth century. The pathologization of homosexuals was made easier by intense state control in the first decades of the twentieth century designed to curb the spread of syphilis and other venereal diseases, including blennorrhea, which were believed to cause degenerations in newborns. As the ideas and practices of positive eugenics spread, the promotion of virility by medical examiners, criminologists, and sexologists concerned with the formation of the character and morphology of boys and men transcended pediatrics and puericulture. The nation should be strengthened by investing in the reproduction of robust and useful citizens.

Since the late 1800s, homosexuals had become symbols of behavior that defied the norms of eugenics for men. Their sexual “passivity” was considered synonymous with impotence and an imbalance of the sexual glands. The presence of “passive pederasts” on the streets of Rio de Janeiro constituted a challenge to the prevailing biological and social order, as their sexual activities were inconsistent with the idea of perpetuating the species through heterosexual biological reproduction, as envisaged by the Getúlio Vargas administration (1930-1945). “Active pederasts” were granted the benefit of anonymity. The book *Homossexualismo e endocrinologia*, by the medical examiner Leonídio Ribeiro, is symptomatic of the social, political, and medical prejudices towards homosexuals throughout Brazilian history. Its interpretation acquires new historical and heuristic relevance when it is examined alongside the circulation of hormonal medicines in the first decades of the twentieth century.

By using biotypology to classify body types, Leonídio Ribeiro conceived of parameters to qualify human sexual development. The science of hormones was useful for him as it enabled treatments to “correct” bodies that differed morphologically from the “ideal.” Opotherapy was chosen as a viable way of leading bodies towards reproductive normality. Endocrinology introduced new assumptions that, added to forensic medicine, criminology, psychology, hygiene, psychiatry, and puericulture, provided different routes of intervention in the bodies of children, adolescents, and adults with the aim of bringing about their eugenic improvement; in other words, keeping alive or restoring the potential to fulfill their biological functions, namely, the reproduction of the species, under the mantle of compulsory virility.

The sheer number of pharmaceutical companies that manufactured glandular extracts for treating male sexual health issues highlights the importance of investing in analyses of the production of scientific knowledge and medical therapies involving the medicalization of male sexuality from the late nineteenth century through the first decades of the twentieth century. From an economic perspective, the range of hormonal extracts indicated for men identified in this study indicates that the incipient pharmaceutical industry supplying synthetic hormones in Brazil in the 1930s saw opotherapeutics made from the organs of non-human animals not so much as a competitor, but as an ally in opening up new markets. Hormonal medications promoting male virility entered the leading Brazilian pharmacies and medical clinics in the 1930s and 1940s. Pansexol, the formula developed by the psychiatrist Antônio Austregésilo, challenged the new synthetic hormone market; indeed, it was advertised in newspapers until 1962. Testicular extracts were prescribed in sexology clinics to correct a range of behaviors considered indicative of male sexual abnormality. Based on the above, the arrests of 195 “passive pederasts” by the Civil Police of Rio de Janeiro, as reported in *Homossexualismo e endocrinologia*, constitute a medical expression of recognition of the legitimacy of the opotherapeutic techniques in circulation in Brazilian society in the first decades of the twentieth century.

## Data Availability

Not deposited in a data repository.

## NOTES

1 Throughout the 1930s, several police raids were carried out with the purpose of repressing homosexuals, samba, and people of African origin (Dias, 2015; Gama, 2012).

2 Translator’s note: In the nineteenth century, the term “pederasty” was used to refer generally to male homosexuality. It and its derivative “pederast” are used in this translation to reflect more closely the nomenclature used in Brazil in the first decades of the twentieth century.

3 On January 14, 1935, Ribeiro was received by Mussolini, in Italy, where he praised the “work of fascism in the protection of motherhood and childhood.” Ribeiro gave a lecture at the Observation Center and Criminal Court of Childhood, where he shared the stage with Mussolini, declaring that “the Italian ideas had largely been followed in the work carried out at the Police Identification Office in the Brazilian capital.” The Italian president “expressed satisfaction upon learning how the Brazilian professor continued the traditions of Italian science in his country” (O Dr. Leonídio…, 14 jan. 1935).

4 In the early 1900s, cretinism was classified as a disease caused by a deficiency of hormones produced by the thyroid. This nosological category was constantly associated with “mongolism,” a racist medical category based on the apparently “mongoloid” facial features of patients from different parts of the world. It was based on theoretical racial atavism and had contradictory effects on the understanding and treatment of these conditions (Löwy, 2017, p.34). The clinical differentiation of these two conditions was facilitated by the introduction of thyroid organotherapeutic extracts as the main therapy. Patients who did not respond clinically to such treatment were diagnosed with mongolism, while patients who did were classified as cretins. The term “mongolism” persisted until 1959, when Down syndrome was definitively identified as a pathology caused by the presence of an extra copy of chromosome 21 in the patient’s body. This clinical condition was later called trisomy 21 (Löwy, 2017, p.34-36). Since then, the term “cretinism” has been associated with the category of “congenital hypothyroidism” and its derivative clinical symptoms, such as myxedema, severe mental retardation, impaired skeletal development, and short stature.
