# Supplementary material for: A proposed cure for homosexuality and the circulation of male hormone therapies in Brazil, 1938-1949
Source: Hist Cienc Saude Manguinhos. 2025 May 19;32:e2025023. doi: 10.1590/S0104-59702025000100023en (PMC12091863; doi:10.1590/S0104-59702025000100023en)
Supplement: Translation

# Translation: A proposta de cura da homossexualidade e a circulação de terapias hormonais masculinas no Brasil, 1938-1949

**DOI:** 10.1590/S0104-59702025000100023

**Authors:** Rodrigo Ramos Lima

**Affiliations:** i Pesquisador de pós-doutorado, Departamento de Medicina Preventiva/Faculdade de Medicina/Universidade de São Paulo. São Paulo – SP – Brasil contatomagisterio@hotmail.com

**Keywords:** Organoterapia, Homossexualidade, Eugenia, Hormônios, Endocrinologia

## Abstract

O artigo analisa a obra *Homossexualismo e endocrinologia* (1938) do médico legista Leonídio Ribeiro. Nela recomenda-se a opoterapia como tratamento de recondução de “pederastas passivos” à heterossexualidade. A opoterapia ou organoterapia, terapêutica criada em fins do século XIX, consistia em tratar humanos doentes das glândulas endócrinas com remédios produzidos a partir de secreções de glândulas de animais não humanos. Examinamos as representações do corpo masculino e o fomento à cultura da virilidade compulsória veiculadas nas propagandas comerciais de extratos glandulares nas décadas de 1930 e 1940 no Brasil. Este estudo visa contribuir para os estudos contemporâneos sobre a história da “cura *gay*” e sua íntima conexão com a eugenia.

Em junho de 1889, o cientista Charles-Edouard Brown-Sécquard (1817-1894), conhecido por seus estudos na área de neurofisiologia, compareceu à Academia Nacional de Medicina de Paris e anunciou que aplicava em si mesmo injeções de extratos testiculares de cachorros e porcos da guiné a título de experiência e que sentia maior disposição e bem-estar. Suas pesquisas visavam comprovar que restaurar a jovialidade e a capacidade criativa dos homens era possível e constituíram-se no marco do nascimento da opoterapia, ou organoterapia, método terapêutico que consistia em utilizar extratos de secreção interna de glândulas de animais não humanos na produção de medicamentos para pacientes com doenças das glândulas endócrinas. Os estudos de Brown-Sécquard tiveram seus métodos questionados por diversos fisiologistas. Além disso, havia dúvidas sobre as glândulas sexuais serem as únicas responsáveis pelo desenvolvimento sadio da sexualidade humana. Apesar disso, o uso dos extratos testiculares foi categorizado como uma terapia de promoção da virilidade e do revigoramento da sexualidade masculina. Para as mulheres, os preparados químicos oriundos de ovários desses mesmos animais eram indicados para histeria, problemas de útero e debilidade devido à idade (Borell, 1976).

O rejuvenescimento se tornou um importante capítulo na fundação da endocrinologia no âmbito global da década de 1920 (Sánchez, 2016; Schlich, 2010). Durante esses anos, o cientista austríaco Eugen Steinach (1861-1944) elaborou a noção de que a interrupção dos canais espermáticos conduzia ao rejuvenescimento dos homens, visto que a liberação de espermas pelos vasos deferentes para o interior do próprio testículo aumentaria a capacidade física e recrudesceria o envelhecimento. As cirurgias de vasectomia tornaram-se sinônimo de absorção das secreções internas testiculares e contribuintes do revigoramento funcional orgânico. O cirurgião francês Serge Voronoff (1866-1951) viajou por diversos países promovendo as técnicas de enxerto de testículos de macacos para os casos de senilidade e perda da virilidade e para a restauração das funções sexuais. Os enxertos viabilizariam uma forma cirúrgica da opoterapia (Sánchez, 2016; Levai, 2016; Cuperschmidt, Campos, 2007). Os efeitos das cirurgias nas glândulas sexuais foram tidos como instrumentos de renovação das forças orgânicas e sexuais dos seres humanos (Sengoopta, 2003). Contudo, as práticas terapêuticas de transplantes de testículos não seguiam o conceito de substituição de órgãos tal qual concebido pelo raciocínio contemporâneo que guia os transplantes de órgãos. Os enxertos de testículos eram utilizados como remédios tanto nas questões sexuais masculinas quanto num variado conjunto de doenças (Schlich, 2010).

Nos últimos anos, pesquisas destacaram as tecnologias hormonais e a opoterapia como objetos que permitem a reflexão histórica da endocrinologia e suas contribuições para a eugenia (Becalossi, 2018, 2020a, 2020b, 2023; Lima, 2021; Logan, 2013; Eraso, 2007; McLaren, 2007, 2012). Na historiografia brasileira, a organoterapia emergiu em estudos relacionados aos temas da produção de bioterápicos (Lima, 2019; Manzoni, 2013; Benchimol, Teixeira, 1993), em estudos de gênero (Rohden, 2009; Lima, 2016), seus usos durante o parto (Mott, 2002; Nucci, Nakano, Teixeira, 2018), promoção de tecnologias contra a impotência (Levai, 2016; Malcher, 2007) e na trajetória da especialização da endocrinologia (Martins, 2001). Segundo a antropóloga Fabíola Rohden (2008), durante a década de 1920, circulou na imprensa leiga uma diversidade de opoterápicos recomendados para o tratamento das funções ovarianas comercializados por laboratórios farmacêuticos brasileiros e internacionais. Para essa autora, a emergência dos hormônios e a identificação do ciclo menstrual nas primeiras décadas do século XX alçaram o ovário à peça central na definição da natureza feminina: sua vitalidade orgânica era capacitar a mulher para a função reprodutiva. Rohden (2008) também sinalizou a inflexão causada pela organoterapia ao entrar na agenda de pesquisas sobre os órgãos reprodutivos femininos, posto que as terapias hormonais levaram as cirurgias de ovariotomia ao ostracismo. Entretanto, seguindo as afirmações de Nelly Oudshoorn (1995) contidas no seu renomado estudo sobre a história dos hormônios na Europa, Rohden (2008, p.147) defendeu, em relação às terapias hormonais masculinas, que teria ocorrido um fracasso na sua comercialização e “na tentativa de criação de uma entidade clínica similar à menopausa”.

O presente artigo objetiva renovar essa interpretação. Como mostra Benninghaus (2012), estudos feministas de gênero na década de 1980, elaborados sob a ótica do construtivismo social, contribuíram para a falta de pesquisas sobre a história da saúde sexual masculina. Chandak Sengoopta (2001) assinalou que, embora os testículos humanos não tenham sido submetidos ao controle médico compulsório tal qual os ovários humanos, é preciso considerar as pesquisas sobre gônadas masculinas em sua própria dimensão clínica.

Neste artigo, examinamos como as indicações terapêuticas para tratamento de homossexuais presentes na obra *Homossexualismo e endocrinologia* (Ribeiro, 1938) permitem investigar as práticas opoterápicas no Brasil. Apesar de a criação dos hormônios sintéticos purificados, na década de 1930, ter contribuído para que muitos organoterápicos deixassem de ser acionados na clínica no cenário internacional, o caso brasileiro demonstra que a situação foi mais favorável à continuidade da venda dos opoterápicos, em virtude da incipiente indústria produtora de hormônios sintéticos. Argumentamos que a circulação das propagandas farmacêuticas dos principais opoterápicos apontados como restauradores da virilidade e da saúde sexual masculina indica que um suposto “fracasso” da venda dos extratos glandulares não se sustenta, dada a prolífica e diversificada comercialização e o uso clínico de extratos testiculares destinados a remediar questões sexuais masculinas no decorrer das décadas de 1930 e 1940.

## Um projeto de cura das homossexualidades

O médico legista Leonídio Ribeiro (1893-1976) alcançou notoriedade no cenário médico científico brasileiro como o primeiro médico a dirigir o Instituto de Identificação da Polícia Civil do Distrito Federal, em 1931. Anexo ao instituto policial, Ribeiro criou o Laboratório de Antropologia Criminal. O periódico *Arquivos de Medicina Legal e Identificação*, publicado e editado pelo médico legista entre 1931-1940, tornou-se a principal plataforma de divulgação dos trabalhos criminológicos realizados por ele e seus colaboradores, como os estudos sobre as relações dos negros com o crime, a pureza de sangue nos índios guaranis e as deformações digitais ocasionadas pela lepra (Dias, 2015; Cunha, 2002).

Em 1938, foi lançado o livro *Homossexualismo e endocrinologia*, como resultado da compilação de textos publicados por Ribeiro no início da década de 1930. Nessa obra, foram inseridas imagens dos detidos e novos capítulos que defendiam o fim da criminalização da homossexualidade em prol da defesa de sua patologização.^1^ Ao longo dos anos, Ribeiro divulgara em revistas de direito e medicina, bem como em conferências em sociedades médicas, conclusões dos seus estudos biotipológicos com os pederastas e isso lhe rendeu – e à sua equipe de pesquisadores – o Prêmio Lombroso^2^ concedido pela Academia Real de Medicina Italiana, consagrando-o, como bem disse Arthur Ramos (citado em Dias, 2015), como o jovem multiplicador da medicina legal no Brasil.

A criminologia positivista do médico italiano Cesare Lombroso (1835-1909) sustentava a tese de que os crimes ocorriam em função da constituição degenerada e atávica dos delinquentes. Lombroso incentivava a investigação dos dados hereditários e dos aspectos morfológicos que indicavam sinais de desequilíbrios orgânicos e patologias motivadoras de atos considerados “anormais” (Dias, 2015). A criminologia positivista possuía natureza pluridisciplinar e a endocrinologia tornou-se o componente teórico que embasava seus argumentos, fornecendo subsídios para a formulação de conceitos como “endocrinopata criminal” (Oliveira Jr., 2012). Ao defender a determinação de traços orgânicos endócrinos como catalisadores de constituições humanas criminosas, Ribeiro figurou como um dos poucos cientistas ainda adeptos do pensamento lombrosiano num momento em que as teses biodeterministas começavam a declinar. Ainda que aceitasse explicações psicanalíticas, Ribeiro reuniu um leque de experiências científicas defensoras do desequilíbrio funcional das glândulas de secreção interna como elemento causador das “inversões sexuais”. Para alcançar a cura dos homossexuais era necessário aplicar a opoterapia.

*Homossexualismo e endocrinologia* (1938) tem prefácio do endocrinologista espanhol Gregório Marañón (1887-1960), principal fornecedor da base teórica e etiológica do termo “homossexualismo” (Dias, 2015). Para Marañón, entre o “varón-tipo” e a “hembra-tipo” – homens e mulheres heterossexuais – havia os fenômenos de intersexualidade e uma variedade de manifestações sexuais que desafiavam a medicina. Segundo esse autor, a ausência de caracteres sexuais bem definidos e de elementos heterossexuais predominantes sinalizavam patologia sexual. Nesse sentido, sexo e sexualidade eram sinônimos. Os critérios de diagnóstico da normalidade do sexo dependeriam da observação dos sinais anatômicos primários – relacionados às genitálias – e secundários – vinculados a características do corpo não ligadas à reprodução, como a distribuição de pelos, a gordura e as medidas do quadril. Os caracteres sexuais funcionais eram sinônimos de interpretação social dos sexos e representavam a biologização dos papéis tradicionais sociais de gênero. Sobre as mulheres recaía a determinação biológica para o instinto da maternidade, a inata sensibilidade aos estímulos afetivos, justificada ausência de habilidades para trabalhos criativos e abstratos, além de resistência passiva e timbre agudo da voz. Os homens destacavam-se pelo instinto de atuação social, defesa da racionalidade e inclinação ao trabalho fora do ambiente doméstico (Ferla, 2004).

Para Marañón, a “perversão do instinto” não é o resultado direto e único da intersexualidade, mas apenas uma predisposição, pois cabe aos fatores ambientais condicionarem a formação da sexualidade do indivíduo. A predisposição seria acordada de seu sono constitucional quando colocada em contato com as primeiras impressões sexuais ocorridas na vida infantil e na crise da puberdade, período no qual as perturbações intersexuais vinham a lume e ocorria a escolha pela normalidade ou anormalidade sexual futura (Ribeiro, 1938). Essa operação conceitual foi beneficiada pelas intersecções entre os saberes endocrinológicos, com o papel do neolamarckismo, em que as influências do meio ambiente na capacidade de alterar a constituição do ser, seja pela ordem hereditária, ou pela força da persuasão hormonal nos comportamentos foi evocada por Eugen Steinach e seus seguidores (Logan, 2013).

Outra referência vital de *Homossexualismo e endocrinologia* foi o endocrinologista italiano Nicola Pende (1880-1970), considerado como principal fundador da biotipologia, ciência responsável por criar categorias dos biotipos humanos (Becalossi, 2020b; MacMillan, 2013; Gomes, 2012). Intimamente atrelada à experiência corporativa do fascismo italiano, a biotipologia concedeu cabedal teórico-biológico para o governo de Benito Mussolini e seus anseios de produzir um “homem novo” (Dias, 2015, p.114).

Em sua obra *Le applicazioni dell’endocrinologia allo studio dei criminali: la scuola positiva*, de 1923, o cientista italiano relaciona endocrinologia e criminologia. Com essa referência, Ribeiro evocou que Pende, diferentemente da percepção difundida na época de que a homossexualidade era resultado apenas da má-formação das glândulas sexuais, considerava-a uma síndrome psíquica e somática resultante do mau funcionamento das glândulas endócrinas. Nessa perspectiva, Ribeiro propôs que a hiperfunção patológica do timo após a puberdade, sobremaneira quando concatenada ao hipertireoidismo constitucional, produziria a homossexualidade. Além dessa proposta etiológica, o médico forense indicou o desequilíbrio funcional das suprarrenais como fator explicativo para os “distúrbios de natureza sexual”, considerando-a como produtora de duas substâncias definidas: a medular e a cortical, a primeira atuando em seu sistema cromafino por meio da adrenalina e a segunda com ação direta na formação dos órgãos genitais (Ribeiro, 1938).

Chiara Becalossi (2018) demonstrou que, durante as atividades do Instituto de Biotipologia em Gênova, e por meio dos principais textos publicados por Nicola Pende na década de 1920, a opoterapia foi elevada a instrumento de correção dos desequilíbrios funcionais das glândulas, que seriam hereditariamente transmitidos. Esses benefícios atribuídos à opoterapia a transformaram em instrumento eugênico. Seguindo a discussão de Lorenzo Benadusi, Becalossi entende que a biotipologia teria, ao menos, dois propósitos eugênicos: por um lado, visava promover a fecundidade e, por outro, aperfeiçoar o estoque populacional italiano por meio do estímulo à virilidade e da repressão do comportamento homossexual.

Repreender o comportamento homossexual foi uma das metas do Estado fascista de Mussolini. Nesses corpos foram empregados a opoterapia, fototerapia, dietas controladas e psicoterapias. Pende descreveu em seus tratados médicos diversas cirurgias de transplantes de glândulas pituitárias, adrenais, tireoide e suprarrenais em pacientes portadores de impotência sexual e neurastenia sexual.

Leonídio Ribeiro, por sua vez, remontou à tradição de experiências com implantações de testículos em acidentados e egressos da guerra com o fito de demonstrar que os pacientes retomavam sua capacidade sexual normal após a aplicação da opoterapia pelo processo de enxertos de testículos:

Há uma observação recente de Dartigues, de Paris, de um nevropata de 33 anos, cujas antigas tendências homossexuais foram logo melhoradas, aparecendo mesmo o desejo sexual e a vontade de casar, dois meses depois da operação de transplantação. Os enxertos ovarianos são hoje, aliás, muito comuns na prática cirúrgica, com excelentes resultados. Há outras observações publicadas, nas quais se obteve o mesmo êxito. ‘Marañón refere três casos em que aconselhou o enxerto, de acordo com a teoria de Voronoff’, sendo que em dois deles as melhoras foram bastante significativas (Ribeiro, 1938, p.171; destaque no original).

Ribeiro ponderou que os enxertos encontravam reservas dentro da própria comunidade científica como método profilático, pois a “opoterapia não é, porém, uma questão definitivamente resolvida, havendo ainda alguns autores que duvidam de suas possibilidades” (Ribeiro, 1938, p.171). A criação de testes padronizados para identificar a eficácia, a segurança e a qualidade dos extratos glandulares foi eclipsada pelo interesse industrial ante os hormônios sintéticos criados no decorrer da década seguinte. Em 1923, Edgar Allen e Edward Doisy haviam anunciado a localização, extração e purificação parcial de hormônios do ovário, denominados estrogênios (Sterling, 2006). Em 1928, os cientistas alemães Selmar Aschheim (1878-1965) e Bernhard Zondek (1891-1966) publicaram o estudo *Immature mouse genital system changes when exposed to pregnant human urine.* Nele, foram relatados o papel fisiológico da foliculina e dos hormônios liberados pelo lobo anterior da hipófise no processo de desenvolvimento das gônadas sexuais. Os cientistas identificaram que os ovos fertilizados pelos espermatozoides liberavam um hormônio denominado gonadotropina coriônica humana (hCG), que atua na liberação de sinais para o útero interromper o ciclo menstrual e preparar a região uterina para a gestação. A partir daí, foram criados os testes de gravidez. Em seguida, os extratos glandulares ovarianos começaram a ser testados em cobaias. O contínuo interesse frente ao potencial comercial desses fármacos resultou na purificação dos hormônios sintéticos femininos, em 1929, obtidos da extração de estrogênios na urina de cavalos e mulheres grávidas.

Em 1931, o bioquímico alemão Adolf Butenandt (1903-1995) obteve cinquenta miligramas de hormônios masculinos a partir de 25 mil litros de urina humana oriunda de quartéis policiais. Quatro anos depois, Ernest Laquer, da indústria europeia Organon, isolou um produto hormonal a partir de testículos de touro que nomeou testosterona. Por conta das dificuldades na obtenção de imensas quantidades desses insumos, a solução foi produzir novos compostos hormonais a partir de elementos orgânicos com estrutura química semelhante. Em 1935, Butenandt, em colaboração com a indústria Schering-Kahlbaum, e Leopold Ruzicka, em parceria com a Ciba Corporation, reportaram, simultaneamente, metodologias que permitiram transformar o colesterol em testosterona sintética. Esses cientistas compartilharam o prêmio Nobel de Química em 1939 (McLaren, 2012; Gaudillière, 2005). Para mensurar a eficácia dos extratos hormonais masculinos foi adotado o crescimento da crista em galos castrados submetidos a injeções de testosterona como método-padrão na aferição da qualidade dos novos sintéticos (Sterling, 2006). No Brasil, as empresas Schering S.A., Ciba Ltda. e Laboratórios Raul Leite compunham a reduzida lista de indústrias que comercializaram hormônios sintéticos masculinos no decorrer da década de 1930.

Cabe registrar que na obra *Homossexualismo e endocrinologia* não há indicações para o uso de hormônios sintéticos masculinos. O endocrinologista espanhol Gregório Marañón, citado no livro, de forma oposta a Leonídio Ribeiro, aconselhou um método terapêutico duplo, composto da associação de enxertos com a opoterapia. Leonídio Ribeiro (1938, p.174) recomendava as cirurgias de enxertos de testículos associada à ingestão de sucos de testículos, preferencialmente frescos, para revitalizar as funções sexuais, além da utilização de “injeções de soro sanguíneo, extraído de animais novos da mesma espécie e, por isso, rico em hormônios testiculares”. O objetivo do tratamento precoce seria diagnosticar a presença de hipogenitalismo, isto é, o subdesenvolvimento dos órgãos genitais, como prova de que a homossexualidade estava vinculada ao pleno desenvolvimento das gônadas sexuais. Numa clara demonstração de apreço pelas técnicas de Serge Voronoff, Ribeiro (1938, p.174) prescreveu:

Os enxertos feitos no homem com a técnica de Voronoff, de glândulas extraídas de macacos superiores, se bem que ainda não completamente eficientes, em todos os casos, demonstram já a possibilidade de resultados favoráveis, em certos indivíduos, especialmente quando realizados precocemente. O próprio Bauer, que assinalou os insucessos de vários autores, nesse sentido, reconhece sua eficácia indiscutível, quando realizados antes da puberdade.

Ribeiro recorreu ao seu mentor profissional, o médico legista Afrânio Peixoto (1876-1947), autor de *Los missexuales* (1931), para justificar seu projeto de cura dos homossexuais. Para Peixoto, o homossexual sofria de uma espécie de tuberculose dupla dos tecidos, a qual era necessário tratar. Com o enxerto “de um dos testículos ectópicos de um jovem; perde os seus gestos invertidos e casa-se” (Ribeiro, 1938, p.93). Outra referência marcante em *Homossexualismo e endocrinologia* foi o argumento evocado pelo criminologista espanhol Jimenez de Ásua (1889-1970) segundo o qual o castigo dado à sexualidade “desviada” era um “absurdo”, posto que a transplantação das glândulas genitais atuava como um “tratamento médico opoterápico bem dirigido” (Ribeiro, 1938, p.55). Durante a Primeira Guerra Mundial, o cientista Eugen Steinach e o urologista Robert Lichtenstern (1874-1952), ambos austríacos, transplantaram o testículo de um homem heterossexual num soldado homossexual de 31 anos acometido pela tuberculose. Doze dias após a cirurgia, o paciente relatou inclinações heterossexuais, seis semanas depois, experimentou a primeira atividade sexual, e logo depois casou-se com uma mulher. Os cientistas relataram, no entanto, que posteriormente o paciente continuou com traços de comportamento considerados afeminados (Schlich, 2010).

Na comunicação apresentada à Sociedade Brasileira de Criminologia, Leonídio Ribeiro (1936) foi indagado pela audiência – composta por médicos e juristas – se as prisões dos homossexuais não seriam uma violência e respondeu que a singularidade de sua pesquisa residia nos registros fotográficos e na análise biotipológica quantitativa dos pederastas passivos, pois sua terapêutica visava somente o corpo do pederasta passivo. Sob o viés da patologização da homossexualidade, James Green (2003, p.209) enfatizou que ao afeminado (bicha) recaía sempre a postura do parceiro “passivo, o que era penetrado”, e sua “passividade” sexual era estigmatizada e associada “a posição social inferior da ‘mulher’”. Por outro lado, o “pederasta ativo”, que assumia o papel público e supostamente privado do homem responsável pela penetração, escapava desses rótulos, pois ao homem “verdadeiro” era concedida a possibilidade de ter relações sexuais com outros homens sem prejuízo do seu *status* social de homem. Afinal, tratar o pederasta ativo e oferecer tratamento para ele poderia ser uma forma de reconhecer a semelhança da masculinidade pederasta ativa ante a heterossexualidade. Em outros termos, a “anormalidade” desses indivíduos não poderia parecer com a normalidade almejada, isto é, os “pederastas ativos” revelariam a semelhança de sua prática sexual penetradora diante do comportamento dos homens heterossexuais (Lima, 2016).

A hermenêutica de *Homossexualismo e endocrinologia* torna-se abrangente quando cotejamos o amplo histórico de interesses médicos e farmacêuticos com os saberes e terapêuticas endocrinológicas em ascensão no Brasil entre finais do século XIX e as primeiras décadas do século XX. Diferente de outros especialistas, Ribeiro não menciona opoterápicos testiculares de sua preferência. Sua obra refletiu considerável predileção pela literatura médica da década de 1920, sobretudo devido à repercussão dos ensinamentos de Voronoff à classe médica brasileira durante sua passagem nas jornadas médicas de 1928 (Levai, 2016; Cuperschmidt, Campos, 2007).

Convém agora resgatar a presença dos produtos opoterápicos utilizados em clínicas médicas e nas sucessivas tentativas de “cura *gay*”. Em seguida, indicamos os principais opoterápicos disponíveis no mercado farmacêutico brasileiro durante as décadas de 1930 e 1940. Observaremos como os papéis sociais esperados para os homens foram representados com riqueza de detalhes nas propagandas dos produtos indicados para tratar impotência, neurastenia e falta de virilidade.

## Extratos glandulares nas prateleiras, manuais e consultórios

Devido à diminuição populacional provocada pela Primeira Guerra Mundial (1914-1918) e pela gripe espanhola (1918), propostas que visavam corrigir o declínio populacional receberam notável adesão. A impotência sexual passou então a ser considerada uma condição clínica que trazia consequências sociais. Além disso, os homens passaram a sofrer a pressão de superar a concorrência das mulheres e dos jovens no mercado de trabalho. Os hormônios sexuais eram enxergados como estimuladores da energia física, psíquica e sexual, e isso assumiu proporções industriais. A eugenia, a endocrinologia e todos os tratamentos acionados para rejuvenescimento da espécie humana representavam o desejo imperativo do século XX de superar os limites biológicos expressos nas noções de envelhecimento, morte e impotência (McLaren, 2007).

Políticas públicas destinadas a aperfeiçoar hábitos de higiene nas gestantes brasileiras foram interpretadas como símbolo de progresso e, sobretudo, como condições de cumprimento da eugenia positiva, representada pela defesa da reprodução biológica da espécie e o fortalecimento da maternidade científica (Stepan, 2005; Freire, 2008; Becalossi, 2023). O discurso endocrinológico estabeleceu a gestação como principal ciclo responsável pela criação daquilo que Renato Kehl (30 ago. 1930) chamou de “patrimônio glandular”, isto é, seio de influências hereditárias hormonais que definiriam aspectos da vida e da personalidade do nascituro. A credibilidade da endocrinologia também aumentou em consequência do sucesso obtido nos tratamentos de casos de cretinismo severo que acometiam crianças.^3^

Com o incremento da produção industrial pública e privada brasileira de extratos glandulares com fins de tratar as desordens sexuais dos corpos masculino e feminino, urgia substituir o excesso sexual como expressão simbólica da constituição do ser brasileiro por experiências sexuais fundadas restritamente no caráter biológico da sexualidade, isto é, ensejar a reprodução da espécie (Oliveira, 2012). Assim, a preocupação com a “homossexualidade” masculina possuía forte sentido eugênico, pois ambicionava promover a virilidade nos homens com a finalidade de que se casassem com mulheres e auxiliassem na reprodução biológica da espécie (Lima, 2016).

O número de publicações brasileiras discutindo as minúcias do comportamento sexual sob o prisma do normal e do patológico cresceu no período entreguerras. Carrara e Russo (2002) destacaram a atuação de dois médicos especialistas classificados como sexólogos possuidores de considerável audiência na comunidade médica e científica brasileira: o gaúcho Hernani de Irajá (1894-1969) e José de Albuquerque (1904-1984), especialista em andrologia. Ambos tiveram contato com as terapias opoterápicas em suas clínicas e posições diferentes sobre esses produtos.

José de Albuquerque ficou conhecido por divulgar nos jornais cariocas serviços especializados de tratamento das questões sexuais masculinas, incluindo a cura da “impotência em moço”. No entanto, o médico colocou-se contrário à utilização desenfreada de produtos opoterápicos (Albuquerque, 12 mar. 1936). Segundo Malcher (2007), o sexologista empreendeu coletas estatísticas da utilização da opoterapia testicular utilizadas para resolver as “sexopatias masculinas” narradas em seu consultório. Entre abril de 1933 e fevereiro de 1935, 90% dos pacientes que o procuravam com queixas de natureza sexual foram orientados a consumir opoterápicos testiculares e prostáticos. Do conjunto de 2.634 pacientes, 1.861 já haviam sido examinados por outros profissionais e, destes, 1.676 já possuíam a receita para adquirir a opoterapia. Para Albuquerque, somente 15% desses pacientes eram aptos à “terapêutica por extratos glandulares”, 75% não tinham necessidade de usar tais produtos e para 10,5% não havia sido indicada a prescrição opoterápica. Albuquerque atuou como oponente dos produtos testiculares e desenvolveu militância a favor da fundação da andrologia no Brasil. No entanto, seu engajamento não foi suficiente para frear o interesse das indústrias produtoras de tais extratos hormonais nem de impedir o acesso da população aos medicamentos.

Hernani de Irajá (1933), por outro lado, em seu *Tratamento dos males sexuais*, reportou arsenais terapêuticos indicados ao tratamento de diversas afecções relacionadas aos quadros sexuais masculino e feminino, seguido de algumas experiências. Irajá elegeu os hormônios orquítico e da próstata como responsáveis pela força viril. Em um dos homossexuais que tratou durante quatro meses, acionou a eletroterapia, extratos testiculares, supra e perirrenais, num total de 120 injeções de hormônio intersticial, acrescido de doses de extratos prostáticos, ginástica respiratória, massagens vibratórias na área da tireoide, tratamento da constipação intestinal, psicanálise e educação da “vontade, mudança de ambiente, leituras de romances de paixões heterossexuais” (Irajá, 1933, p.212). Mesmo registrando fracasso nas tentativas de cura – dos 93 casos, alegou sucesso somente em 12 –, a esperança de Irajá em curar os homossexuais permanecia, visto que um médico de sua confiança havia curado uma lésbica com extratos de tireoide (p.255).

Além do aparato hormonal, Irajá (1933, p.29) reforçou o papel dos alimentos no tratamento da impotência e enfatizou que eram raros os homossexuais que queriam se curar. Os “tentados a sodomia passiva” ou “pederastia de compensação”, como marinheiros, soldados e internos, deveriam buscar a “reeducação heterossexual” e o desenvolvimento da estética pelo sexo oposto por meio da pintura e da escultura, além do afastamento “dos centros onde se tenha iniciado e desenvolvido o pendor”, somado de “educação moral e cívica, psicanálise, eletroterapia e cirurgia”. O apreço pela eletroterapia em sua fórmula terapêutica também foi registrado, pois “grandes sessões em série longa de galvanização positiva e alta frequência embotaram a hipersensibilidade anurrectal de um rapaz de 16 anos que praticava a pederastia passiva pela alta erogeneidade da região com prejuízo das zonas eretógenas comuns em seu sexo. Não existiam parasitas intestinais” (Irajá, 1933, p.147). De acordo com o médico, o paciente procurou a clínica espontaneamente e foi “curando-se de um modo completo”.

Em outra passagem, o apelo à observação criteriosa dos corpos infantis também foi indicado, pois em casos de “inversões constitucionais sem transformação dos caracteres sexuais primários ou secundários” é de bom aviso observar as crianças desde três ou quatro anos”. Os pais deveriam evitar que os meninos entrassem em contato com brincadeiras do “sexo contrário”, e o mesmo foi dito sobre as meninas (Irajá, 1933, p.148). Na segunda edição da obra *Tratamento dos males sexuais*, Irajá (1937) descreveu os sintomas clínicos da distrofia testicular manifesta, caracterizada por “amolecimento dos testículos, atonia dos escrotos, falta de ereção ou ereção incompleta com grandes veias nos pênis”, e afirmou empregar os sucos de testículos, a diatermia peniana e testicular com eletrodos especiais, raios infravermelhos e ultravioletas, termoterapia e o método de ventosas sexuais. Além desses, Irajá ressaltou ser importante suplementar o tratamento com injeções do “Pansexol (M)” do Professor Austregésilo e as substâncias Anterofisina e Cortical dos “Hormônios Sexuais – masculino S.A.”, Testogan, entre outros (Irajá, 1937, p.173).

A segunda edição também reportou o tratamento de cinco estudos de caso de pacientes com “inversão sexual”: três homens e duas mulheres. O jovem A.F., de 21 anos, possuía ginecomastia e “inversão sexual”. Seu tratamento consistiu na aplicação de estimulações elétricas galvânicas cujo polo negativo foi inserido no pênis, e o positivo, no ânus, 120 injeções de extratos testiculares e prostáticos associados com ginástica, massagens na região tireoideana e psicanálise durante quatro meses. Irajá admitiu que a tentativa de cura de A.F. falhou (Becalossi, 2020b, p.19).

O segundo caso de inversão sexual masculina foi de um paciente de 29 anos, com constituição “normotipo atlética”, pelos no corpo, voz masculina e glândulas sexuais normais, mas que desde criança vestia roupas das irmãs. Durante três meses de tratamento ininterrupto, o jovem recebeu 51 tratamentos hormonais, combinados com psicanálise e hipnose. Para Irajá, os resultados foram animadores porque, apesar de o rapaz continuar sentindo desejo por homens, passou a praticar relações sexuais com mulheres. O terceiro caso de “inversão sexual masculina” é de um homem de 68 anos, portador de constituição “quase normal” que, apesar das crises de hemorroidas, não conseguia evitar a prática de sexo com homens. O tratamento aplicado consistiu em banhos das genitálias aos 45 graus duas vezes ao dia, somados à exposição de luz ultravioleta e estimulação elétrica galvânica das zonas erógenas. Por três meses, o paciente foi submetido aos extratos testiculares, prostáticos e do córtex renal. Novamente, houve fracasso na cura do paciente (Irajá, 1937).

Entre as pacientes havia L.S., de 25 anos, com “inversão sexual” feminina que apresentava atitudes do tipo masculino, como inclinação aos negócios e matemática. Os caracteres sexuais secundários femininos não haviam se desenvolvido. Desde os 10 anos sentia atração sexual por mulheres, iniciando as práticas sexuais ativas e passivas com elas aos 17 anos. L.S. foi submetida a quarenta injeções de “gynhormon 50 u.r.” e vinte ampolas de hipófises. A paciente ganhou peso com esse tratamento, mas os desejos homossexuais permaneceram. Posteriormente, Irajá inseriu a aplicação diatérmica diária nas ancas para estimular os ovários.

Outra paciente, de 40 anos, iniciou sua vida sexual com diversas mulheres aos 18 anos. A primeira fase de tratamento incluiu o uso de 15 injeções de extratos tireoidianos. Em seguida, foram acionadas trinta novas aplicações desse hormônio somadas a um regime de descanso intensivo. Em face das melhoras na condição corporal, Irajá introduziu os extratos testiculares no tratamento. Novamente, as inclinações homossexuais da paciente permaneceram. Segundo Becalossi (2020b, p.19), Irajá registrou ser cético ante o resultado das terapias hormonais, pois constatava melhoramentos no corpo dos pacientes que não eram acompanhados do desaparecimento dos desejos homossexuais.

A neurastenia sexual também foi acionada para enquadrar o comportamento sexual desviante. Concebida pelo médico neurologista nova-iorquino Georges Miller Beard (1838-1883), em 1869, essa patologia e seu quadro clínico compreendiam uma série de sintomas, tais como a exaustão geral, sensibilidade da coluna e hiperestesia geral, dormências localizadas e periféricas, dores vagas e neuralgias, paralisias temporárias, distúrbios gástricos, cefaleias, problemas no sono, pressão na cabeça, controle mental deficiente, problemas na concentração, irritação mental, fobias, transtornos sexuais nos homens, como ejaculação involuntária de sêmen, impotência parcial ou completa, irritações na uretra e, nas mulheres, deslocamento dos órgãos, inflamações, irritações no útero e nos ovários (Zorzanelli, 2009).

Um bom exemplo da utilização da neurastenia como categoria diagnóstica pode ser encontrado em *Psiconeuroses e sexualidade: a neurastenia sexual e seu tratamento*, de 1919, do prestigiado psiquiatra Antônio Austregésilo (1876-1960), a quem Irajá dedicou a publicação de sua obra. Nessa obra, Austregésilo (1919) recomendou a planta africana ioimbina associada com estricnina, ácido fosfórico e os extratos testiculares, conhecidos no mercado como “sequardina, espermina de Poehl, Orchidan”, para o tratamento das enfermidades sexuais dos homens. Com essa guia prescritiva, Austregésilo criou a fórmula do Pansexol, um novo extrato produzido com extratos de testículos de touro comercializado sob a instigante chamada “Com o rejuvenescimento glandular, seremos eternamente jovens”. A presença do Pansexol nas páginas da imprensa (Figura 1) no pós-ascensão dos hormônios sintéticos permite inferir que a emergência desses novos hormônios não encerrou de forma abrupta a disponibilidade dos produtos opoterápicos. Esse produto foi o único extrato glandular composto por órgãos de animais cuja propaganda comercial circulou na imprensa carioca até 1962.

**Figura 1:** : Propaganda do Pansexol (Com o rejuvenescimento…, 10 abr. 1947)

Destaca-se também o extrato glandular elaborado pelo sexólogo alemão Magnus Hirschfeld (1868-1935) e o andrologista Bernhard Schapiro (1888-1966). Inicialmente vendido como Testifortan, esse extrato foi responsável por boa parte das receitas do Institute for Sexual Science e, a partir de 1929, passou a ser comercializado com o nome de Pérola Titus (McLaren, 2007). Sua circulação no Brasil foi expressiva durante o decorrer dos anos 1930, visto que foi propagandeado nos mais diversos periódicos da imprensa leiga na capital federal e em diversos estados do país. Além de fazer referência ao “Famoso Instituto de Berlim”, seus anúncios exibiam *designs* arrojados que variavam constantemente. Os conteúdos informativos sobre o produto produziam uma sequência de textos persuasivos sobre “insuficiência ou distúrbio sexual” masculinos. Composto por extratos testiculares associados com hipófise e suprarrenais, o preparado Pérola Titus propunha restaurar as funções glandulares masculinas e restabelecer “suas possibilidades normais de perpetuador da espécie” (Para os grandes…, 28 maio 1933).

Os anunciantes da Pérola Titus valorizavam os heróis, sua força e virilidade, pois “todos os homens deveriam ser abnegados, corajosos e heroicos”. Para alcançar esse patamar, os homens deveriam ter “um sistema glandular endócrino perfeito” (Heróis, set. 1935, p.3). Para esses anunciantes, um jovem que portava disfunções endócrinas poderia ser considerado “um velho para todos os efeitos”, enquanto o velho portador de “glândulas ativas e equilibradas” poderia ser considerado um “homem praticamente jovem”. O foco das propagandas era alertar para os sinais da neurastenia como “caráter incerto, eruptivo e tempestuoso” e que oscilavam com traços de mansidão, inércia, cansaço, astenia sexual e serenidade (O mal…, set. 1941).

A propaganda invocava ainda o sentimento político local e global do período, expresso pelo crescimento dos movimentos integralistas, partidos de extrema direita e intensa valorização do militarismo. Na campanha *Integralismo e ditadura sexual* (Figura 2) foi destacado que, para “vencer” este clima de “desmedida agitação”, os homens deveriam ter glândulas de secreção interna em harmonia. Tendo em vista que em todos os ambientes predominava “a campanha de seleção” onde os países “exigem que seus filhos sejam fortes”, a propaganda servia como uma espécie de convocação ao alistamento hormonal.

**Figura 2:** : Anúncio de Pérolas Titus (Integralismo, 19 set. 1933)

Pelas farmácias cariocas também circularam as Drágeas Hormônicas veiculadas como “Um golpe nas neurastenias”. Esse extrato, produzido pelo professor italiano Francisco Figari da Real Universidade de Gênova, compunha-se de fósforo orgânico e hormônios de “certas glândulas de secreção interna”, com capacidade de reparar os danos causados pela neurastenia sexual devido ao seu poder restaurador “mais eficiente, quiçá mais estável, do que o famoso enxerto preconizado por Voronoff” (Drágeas…, 6 jan. 1937). Os consumidores que desejassem conhecer os pormenores das drágeas hormônicas poderiam adquirir a monografia sobre o extrato, distribuída gratuitamente no “Departamento de Difusão da Neoterapia Científica, na rua do Ouvidor”.

Igualmente destinado ao tratamento das debilidades glandulares, fizeram-se presentes nas farmácias e na imprensa fluminense as Gotas Mendelianas. Seu compromisso era ativar as glândulas “germinadoras do homem e nos ovários da mulher” e atuar no estímulo do sistema nervoso. Os anunciantes registraram que o medicamento não afetava o coração e era ofertado como produto “valioso” e “insubstituível” para aqueles que careciam de “um tônico nervino enérgico e rápido”. Os males orgânicos seriam dissipados como por “encanto”, e as Gotas Mendelianas dariam “nova vida e coragem aos velhos neurastênicos, tornando-os fortes e potentes” (Gotas…, 29 nov. 1938).

Nas farmácias também se comercializava o Glantona (Figura 3), extrato glandular composto por testículos de touro ao qual se atribuía a capacidade de curar as perturbações sexuais como impotência, neurastenia sexual, senilidade precoce, debilidades físicas e mental, além de combater o esgotamento e o envelhecimento prematuro, permitindo conservar e recuperar o vigor da mocidade pela alimentação de certas glândulas associadas à esfera reprodutiva (Fraqueza…, 14 jun. 1937).

**Figura 3:** : Anúncio de Glantona (Não existe…, 5 mar. 1949)

No início da década de 1930, os hormônios sintéticos purificados passaram a ser comercializados no Brasil pelas multinacionais Schering, Ciba Ltda. e pela companhia brasileira Laboratórios Raul Leite. Os novos fármacos sintéticos conquistaram paulatinamente as páginas de periódicos médicos especializados e da imprensa leiga e ganharam força na indústria farmacêutica brasileira entre as décadas de 1940 e 1960, em função de seu uso clínico no tratamento da esterilidade reprodutiva de homens e mulheres e em resposta à demanda por anticoncepcionais femininos (Lima, 2021; Bonan, Teixeira, Nakano, 2017).

Apesar do esforço militante de José de Albuquerque contra as práticas opoterápicas e o emergente discurso a favor dos hormônios sintéticos por endocrinologistas e fisiologistas, as recomendações opoterápicas testiculares permaneceram presentes na imprensa leiga e nos manuais médicos que tratavam de problemas sexuais masculinos no decorrer das décadas de 1930 e 1940. Outrossim, mais do que revelar o imaginário da indústria farmacêutica sobre o comportamento social, político e biológico esperado para os homens, as propagandas farmacêuticas expressavam que as disfunções glandulares não deveriam impedir o usufruto das funções orgânicas masculinas, sendo as atividades sexuais e o revigoramento da saúde compromissos que os homens deveriam garantir a si mesmos. A atmosfera eugênica de promoção da atividade reprodutora da espécie e a contribuição da endocrinologia para o cuidado das saúdes sexuais masculina e feminina estimularam a diversificada oferta de extratos glandulares em diferentes estados brasileiros.

## Considerações finais

A dinâmica de prevenção, controle e repressão da sexualidade masculina desviante adentrou no horizonte das políticas de saúde pública brasileira no alvorecer do século XX. O processo de patologização dos homossexuais foi beneficiado pelo intenso controle estatal brasileiro das primeiras décadas do século XX em relação à sífilis e a outras doenças venéreas como a blenorragia, consideradas patologias catalizadoras da degeneração em neonatos. Influenciada por uma atmosfera de ideias e práticas da eugenia positiva, a promoção da virilidade por médicos-legistas, criminologistas e sexólogos preocupados com a formação do caráter e da morfologia corporal dos meninos e homens da nação brasileira transcendeu os ambientes pediátricos e da puericultura. Urgia fortalecer a nação sob as bases do investimento na reprodução de cidadãos robustos e úteis ao país.

Desde finais do século XIX, os homossexuais tornaram-se emblema do comportamento masculino avesso à norma eugênica. Sua “passividade” no ato sexual era considerada sinônimo de impotência e desequilíbrio das glândulas sexuais. A presença dos “pederastas passivos” nas ruas cariocas desafiou as ordens social e biológica predominantes, pois suas atividades sexuais escapavam do projeto de perpetuação da espécie por meio da reprodução biológica de cunho heterossexual almejada pelo Estado varguista. Ao pederasta ativo foi concedido o benefício do anonimato. A obra *Homossexualismo e endocrinologia*, do médico legista Leonídio Ribeiro, é expressão sintomática da permanência secular dos preconceitos sociais, políticos e médicos quanto aos homossexuais na história brasileira. Sua interpretação adquire maior relevância histórica e heurística quando examinada em consonância com o cenário da circulação de medicamentos hormonais disponíveis nas primeiras décadas do século XX.

Ao acionar a biotipologia como metodologia qualificadora dos biotipos corporais, Leonídio Ribeiro preconizou parâmetros para qualificar o desenvolvimento sexual humano. A ciência dos hormônios lhe serviu de apoio, pois instituiu terapêuticas atuantes na correção dos corpos com aspectos morfológicos destoantes dos “tipos ideais”. A opoterapia foi escolhida como terapêutica viável para o processo de condução dos corpos à normalidade biológica reprodutiva. A endocrinologia trouxe novos pressupostos que, somados à medicina legal, criminologia, psicologia, higiene, psiquiatria e puericultura, legaram diferentes rotas de intervenção sobre o corpo das crianças, adolescentes, adultos e senhores com fins de inserir neles o aperfeiçoamento eugênico constante, isto é, manter vivo no horizonte de suas vidas a necessidade de concretização de suas funções biológicas ancoradas na reprodução da espécie sob o manto da virilidade compulsória.

A diversidade de empresas farmacêuticas fornecedoras de extratos glandulares indicados ao tratamento da saúde sexual masculina ressalta a relevância de investirmos em análises sobre a produção de conhecimentos científicos e terapêuticas médicas relacionadas à medicalização das questões sexuais masculinas de finais do século XIX e primeiras décadas do século XX. Do ponto de vista econômico, a identificação de uma diversidade de extratos hormonais indicados aos homens, relatados neste estudo, permite considerar que a incipiente indústria farmacêutica fornecedora de hormônios sintéticos, presentes no Brasil durante a década de 1930, viu nos opoterápicos elaborados com órgãos de animais não humanos antes um aliado que abria espaços do que um inimigo a ser combatido. A promoção da virilidade masculina adentrou no escopo dos medicamentos hormonais existentes nas principais farmácias e clínicas médicas brasileiras nas décadas de 1930 e 1940. A fórmula do Pansexol do psiquiatra Antônio Austregésilo desafiou o novo mercado de hormônios sintéticos. Suas propagandas circularam na imprensa leiga até 1962. Os extratos testiculares foram prescritos em clínicas de sexologia para corrigir uma série de comportamentos considerados expressões da anormalidade sexual masculina. Pelo exposto, as prisões dos 195 “pederastas passivos” pela Polícia Civil do Rio de Janeiro, relatadas na obra *Homossexualismo e endocrinologia*, constituem-se como expressões médicas de reconhecimento da legitimidade das técnicas opoterápicas em circulação na sociedade brasileira nas primeiras décadas do século XX.

## Data Availability

Não estão em repositório digital.

## NOTAS

1 Ao longo dos anos 1930, diversas operações policiais foram realizadas com a finalidade de perseguir homossexuais, o samba e os povos de matriz africana (Dias, 2015; Gama, 2012).

2 Em 14 de janeiro de 1935, Ribeiro foi recebido pelo presidente Mussolini na Itália, onde elogiou a “obra do fascismo no domínio da proteção à maternidade e à infância”. Ribeiro realizou uma palestra no Centro de Observação e Tribunal Criminal da Infância, onde dividiu a fala com Mussolini, e declarou que “as ideias gerais italianas tinham sido seguidas nos trabalhos realizados no Gabinete de Identificação da Polícia da capital brasileira”. O presidente italiano “expressou a satisfação que sentia ao saber como o professor brasileiro continuava no seu país as tradições da ciência italiana” (O Dr. Leonídio…, 14 jan. 1935).

3 No início do século XX, o cretinismo foi enquadrado como uma doença ocasionada pela insuficiência dos hormônios produzidos pela tireoide. Essa categoria nosológica esteve constantemente associada ao “mongolismo”, que foi uma categoria médica preconceituosa, baseada nos traços faciais asiáticos presentes em pacientes habitantes de diversas regiões do mundo e que apresentavam traços “mongólicos”. Ela foi baseada num atavismo racial teórico e trouxe efeitos contraditórios para o entendimento e a abordagem terapêutica dessas condições (Löwy, 2017, p.34). A diferenciação clínica desses dois quadros foi facilitada pela introdução dos extratos organoterápicos tireoidianos como principal terapêutica. Os pacientes sem evolução clínica eram diagnosticados com mongolismo, enquanto os pacientes com melhoras clínicas eram classificados como cretinos. O termo mongolismo perdurou até 1959, quando a síndrome de Down foi definitivamente identificada como uma patologia resultante da presença adicional de uma cópia do cromossomo 21 no organismo do paciente. Essa condição clínica foi posteriormente chamada de trissomia 21 (Löwy, 2017, p.34-36). Desde então, o termo cretinismo foi associado à categoria “hipotireoidismo congênito” e seus derivados sintomas clínicos como mixedema, retardo mental grave, desenvolvimento esquelético prejudicado e estatura baixa.
